# Total Antioxidant and Oxidative Status as Potential Biomarkers of Alcohol Overdose

**DOI:** 10.3390/ijms26010082

**Published:** 2024-12-25

**Authors:** Iwona Ptaszyńska-Sarosiek, Edyta Gołaś, Miłosz Nesterowicz, Anna Niemcunowicz-Janica, Anna Zalewska, Małgorzata Żendzian-Piotrowska, Mateusz Maciejczyk

**Affiliations:** 1 Department of Forensic Medicine, Medical University of Bialystok, 15-269 Bialystok, Poland; iwona.ptaszynska-sarosiek@umb.edu.pl (I.P.-S.); annjanica@gmail.com (A.N.-J.); 2 Students Scientific Club “Biochemistry of Civilization Diseases” at the Department of Hygiene, Epidemiology and Ergonomics, Medical University of Bialystok, 15-269 Bialystok, Poland; edyta.golas@sd.umb.edu.pl (E.G.); milosz.nesterowicz@gmail.com (M.N.); 3 Department of Restorative Dentistry, Medical University of Bialystok, 15-269 Bialystok, Poland; azalewska426@gmail.com; 4 Department of Hygiene, Epidemiology, and Ergonomics, Medical University of Bialystok, 15-269 Bialystok, Poland; mzpiotrowska@gmail.com

**Keywords:** alcohol toxicity, alcohol dependence, alcohol poisoning, redox biomarkers, total antioxidant capacity, total oxidative status, oxidative stress

## Abstract

Serious alcohol-associated hazards underscore the need to develop new biomarkers reflecting the biological changes caused by chronic alcohol use and predicting the risk of alcohol-related death. Oxidative stress is one mechanism of alcohol toxicity. The blood and urine redox status (total antioxidant capacity [TAC], total oxidative status [TOS], and oxidative stress index [OSI]) was assessed in 105 people who died a sudden death (controls), 47 people who died of alcohol overdose, and 102 people with alcohol dependency. TAC and TOS were determined utilizing the colorimetric method. Non-parametric tests were used for statistical analysis. Blood and urine TAC levels were significantly elevated in individuals both with alcohol dependency and alcohol poisoning compared with controls. TOS levels were elevated in the blood of both study groups compared with the control group, and significantly higher in patients with alcohol dependency compared with the group with alcohol poisoning. TAC in the blood highly correlated with blood alcohol content. Receiver operating characteristic (ROC) analysis showed that the blood TAC effectively discriminated between individuals with alcohol poisoning and alcohol dependency with high sensitivity and specificity. Our study confirmed impaired redox homeostasis in people with alcoholism and indicated the utility of TAC, TOS, and OSI as biomarkers of alcohol exposure.

## 1. Introduction

Currently, 2.5 billion people around the world drink alcohol, with an average consumption of 27 g per day. A large number of those people (264 million men and 136 million women) suffer from alcohol use disorders (AUD) [1]. Alcohol addiction is a chronic progressive disorder characterized by an inability to regulate alcohol consumption [2]. That condition has profound consequences for physical and mental health, affecting virtually every organ system in the body [3]. One of the critical pathological effects of alcohol exposure is its impact on redox homeostasis. Alcohol metabolism generates reactive oxygen species (ROS), impairing cell metabolism and gene expression by proteins, lipids, and DNA oxidation [4]. Constant oxidative stress contributes to the pathogenesis of alcohol-related diseases, including liver cirrhosis, cardiovascular disease, and neurodegenerative disorders [2,5]. In severe cases, the cumulative damage caused by oxidative stress may lead to fatal alcohol poisoning, making alcoholism a significant cause of preventable death worldwide [6,7].

The severe health hazards associated with alcoholism underscore the importance of finding reliable biomarkers that may indicate alcohol dependence and predict the risk of alcohol-related death. Effective biomarkers would allow for earlier diagnosis, more targeted intervention, and potentially more effective treatment for people with AUD. Such biomarkers are being sought, aiming to identify indicators that reflect the biological changes associated with chronic alcohol use and its complications [8,9]. Given the vital impact of alcohol on systemic redox homeostasis, total antioxidant capacity (TAC) and total oxidative status (TOS) may be potential diagnostic biomarkers [10,11,12]. The body’s antioxidant defense is composed of several antioxidant enzymes (e.g., catalase [CAT], glutathione peroxidase [GPx], and superoxide dismutase [SOD]) and non-enzymatic antioxidants such as uric acid (UA), thiol groups, and antioxidant vitamins (vitamin A, C, D, and E). Oxidative stress indicators are the products of oxidative damage to proteins, lipids, and DNA, which complicates the assessment of redox homeostasis. Therefore, a reliable approach may be to evaluate the circulating TAC and TOS. TAC measures the total ability of antioxidants, providing an overall picture of the body’s antioxidant systems [13,14]. TOS quantifies the total amount of oxidants, reflecting the oxidative load in the body [10,12,15]. The oxidative stress index (OSI) is a ratio of TOS to TAC, offering a comprehensive assessment of redox homeostasis, including oxidant and antioxidant components [11,12].

Previous studies have shown a systemic redox imbalance in patients with AUD, including disturbances in blood TAC and TOS [16,17,18,19,20,21,22,23]. To date, however, circulating redox status has not been assessed in patients with alcohol dependency compared with those who had died of alcohol overdose. In our pilot study, we showed a significantly higher TAC in the urine of 22 people who had died due to acute ethanol intoxication compared with people who had died suddenly. This study aims to investigate the efficacy of TAC, TOS, and OSI as biomarkers of alcohol overdose. For this purpose, we have assessed redox homeostasis on a larger cohort consisting of 47 people who had died of alcohol overdose and 105 people who had died a sudden death. In addition, we also included a group of 102 individuals with alcohol dependency. By assessing TAC, TOS, and OSI in blood and urine, we sought to understand their potential for indicating the presence of AUD and predicting the risk of fatal outcomes associated with chronic alcohol abuse. This research may improve diagnostic and prognostic tools in the clinical management of alcoholism, ultimately contributing to better health outcomes.

## 2. Results

### 2.1. Blood Redox Status

**Figure 1:**
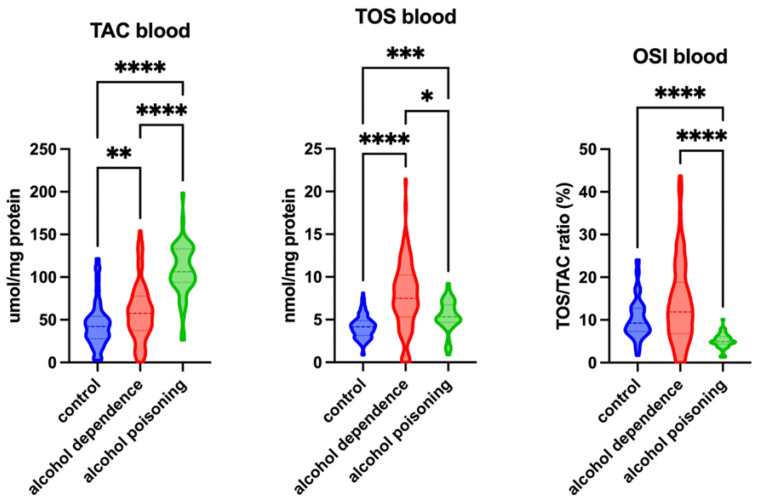
Redox status in the blood of the control group, patients with alcohol dependency, and patients with alcohol poisoning. Abbreviations: TAC—total antioxidant capacity; TOS—total oxidative status; OSI—oxidative stress index. Differences statistically significant at * <0.05, ** <0.01, *** <0.001, and **** <0.0001.

In this study, we have shown that TAC levels in the blood were significantly higher in the groups with alcohol dependency and alcohol poisoning (*p* = 0.0042 and *p* < 0.0001, respectively) compared with the control group. Moreover, TAC levels were significantly higher in the group with alcohol poisoning (*p* < 0.0001) compared with the patients with alcohol dependency. Similarly, the blood TOS levels were significantly higher in the groups with alcohol dependency and alcohol poisoning (*p* < 0.0001 and *p* = 0.0006, respectively) compared with the control group. However, TOS levels were significantly lower in the group with alcohol poisoning (*p* = 0.0108) compared with the patients with alcohol dependency. The OSI index was significantly lower in the blood of the patients with alcohol poisoning compared with the alcohol-dependent and control groups (*p* < 0.0001 and *p* < 0.0001, respectively) (Figure 1).

Receiver operating characteristic (ROC) analysis was conducted to evaluate redox biomarkers’ diagnostic value in the blood. A blood TAC concentration above 86.2 μmol/mg allowed to differentiate between patients with alcohol poisoning and people in the control group. The area under the curve (AUC) for TAC was 0.92. Similarly, TAC levels in blood above 88.11 μmol/mg of protein, with sensitivity and specificity of 82.98% and 83.84%, respectively, facilitated the differentiation between patients with alcohol poisoning and alcohol dependency. A blood TOS content above 5.34 μmol/mg of protein allowed for distinguishing between the alcohol-dependent and control groups. In turn, measuring the TOS concentration in blood, with sensitivity and specificity of 72.34% and 70.59%, respectively, allowed for differentiation between the patients with alcohol poisoning and the control group. Furthermore, measuring the TOS in blood, with 68.09% sensitivity and 66.34% specificity, allowed for the differentiation between the patients with alcohol poisoning and alcohol dependency. A blood OSI concentration below 6.32 μmol/mg allowed for the distinction between the alcohol-poisoned and control groups. Similarly, OSI levels in the blood below 6.48 μmol/mg of protein, with sensitivity and specificity of 84.78% and 86.02%, respectively, facilitated the differentiation between the patients with alcohol poisoning and the alcohol-dependent group. The AUC for the OSI was 0.89 (Table 1).

Depending on the alcohol concentration in the blood, there are specific symptoms of alcohol poisoning. We, therefore, have compared redox status to alcohol concentration and divided the participants into four groups. At concentrations of 1.1–2‰, disturbances of balance, agility, and movement coordination, reduced intellectual performance, delayed reaction time, irritability, aggressive behavior, sexual agitation, increased blood pressure, and heart rate were observed. At concentrations of 2.1–3‰, gibberish speech, body imbalance, increased drowsiness, significantly reduced ability to control one’s behavior, and slowing down were noted. At concentrations of 3.1–4‰, blood pressure drops, body temperature decreases, physiological reflexes weakened or absent, and profound disturbances in consciousness may lead to a coma. People with a blood alcohol concentration above 4‰ are found to be in a deep coma or experiencing respiratory and vasomotor center dysfunction [24,25].

**Figure 2:**
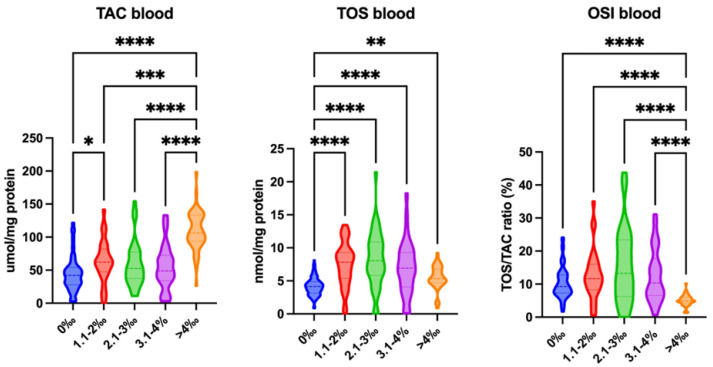
Redox status in blood of subjects with varied blood alcohol concentrations: 0‰, 1.1–2‰, 2.1–3‰, 3.1–4‰, and more than 4‰. Abbreviations: TAC—total antioxidant capacity; TOS—total oxidative status; OSI—oxidative stress index. Differences statistically significant at * <0.05, ** <0.01, *** <0.001, and **** <0.0001.

We have demonstrated that TAC levels were significantly higher in all patients with a blood alcohol concentration near 1.1–2‰ (*p* = 0.0295) compared with the control group. Moreover, TAC levels were significantly higher in patients with a blood alcohol concentration above 4‰ compared with patients with a blood alcohol concentration below 4‰ (>4‰ vs. 0‰: *p* < 0.0001; >4‰ vs. 1.1–2‰: *p* = 0.0002; >4‰ vs. 2.1–3‰: *p* < 0.0001; >4‰ vs. 3.1–4‰: *p* < 0.0001). TOS levels were significantly higher in all patients with a blood alcohol concentration equal/greater than 1.1‰ compared with the control group (1.1–2‰ vs. 0‰: *p* < 0.0001; 2.1–3‰ vs. 0‰: *p* < 0.0001; 3.1–4‰ vs. 0‰: *p* < 0.0001; >4‰ vs. 0‰: *p* = 0.001). The OSI was significantly lower in patients with a blood alcohol concentration higher than 4‰ compared with patients with a blood alcohol concentration below 4‰ (>4‰ vs. 0‰: *p* < 0.0001; >4‰ vs. 1.1–2‰: *p* < 0.0001; >4‰ vs. 2.1–3‰: *p* < 0.0001; >4‰ vs. 3.1–4‰: *p* < 0.0001) (Figure 2).

A blood TAC concentration above 85.16 μmol/mg of protein with high sensitivity and specificity (86.96% and 85.71%, respectively) allowed for the differentiation between patients with a blood alcohol content above 4‰ and those with 3.1–4‰. The AUC for TAC was 0.89. Similarly, measuring the OSI with 82.22% sensitivity and 80.77% specificity allowed for the differentiation between patients with a blood alcohol content above 4‰ and those with 3.1–4‰. The AUC for the OSI was 0.85. Moreover, the blood TOS content above 5.1 μmol/mg of protein allowed to distinguish between the patients with a blood alcohol content approximating 1.1–2‰ and those with 0‰ (Table 2).

### 2.2. Urine Redox Status

**Figure 3:**
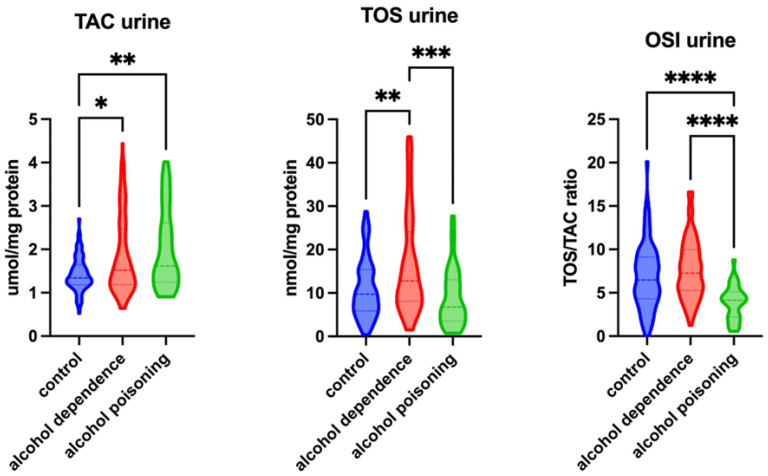
Redox status in the urine of the control group, patients with alcohol dependency, and patients with alcohol poisoning. Abbreviations: TAC—total antioxidant capacity; TOS—total oxidative status; OSI—oxidative stress index. Differences statistically significant at * <0.05, ** <0.01, *** <0.001, and **** <0.0001.

TAC levels in the urine were significantly higher in the alcohol-dependent and alcohol-poisoned groups compared with the control subjects (*p* = 0.0333 and *p* = 0.0089, respectively). Moreover, TOS levels were significantly higher in patients with alcohol dependency compared with the control group (*p* = 0.0035). TOS levels were also considerably lower in the alcohol-poisoned group (*p* = 0.0002) compared with the alcohol-dependent group. The OSI was significantly lower in the urine of patients with alcohol poisoning compared with the alcohol-dependent and control groups (*p* < 0.0001 and *p* < 0.0001, respectively) (Figure 3).

The urinary TOS content below 10.26 μmol/mg of protein allowed for the differentiation between patients with alcohol poisoning and alcohol dependency. The AUC for TOS was 0.72. Measuring the urinary OSI, with AUC values of 0.76, allowed to distinguish the alcohol-poisoned group from the control group. Similarly, the urine OSI concentration below 5.22 μmol/mg allowed us to differentiate the patients with alcohol poisoning from the alcohol-dependent group. The AUC for the OSI was 0.84 (Table 3).

**Figure 4:**
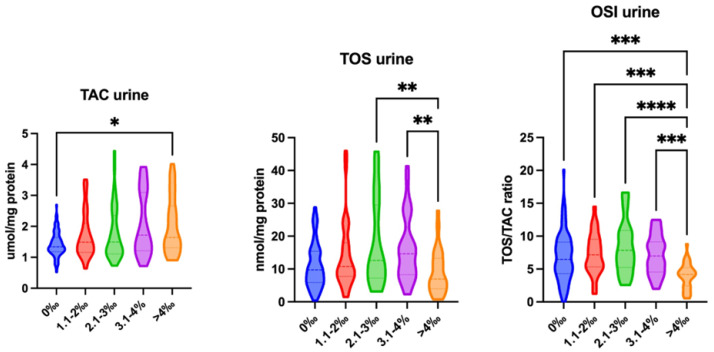
Redox status in the urine of patients with varied blood alcohol concentrations: 0‰, 1.1–2‰, 2.1–3‰, 3.1–4‰, and more than 4‰. Abbreviations: TAC—total antioxidant capacity; TOS—total oxidative status; OSI—oxidative stress index. Differences are statistically significant at * <0.05, ** <0.01, *** <0.001, and **** <0.0001.

Redox status in the urine was also dependent on the blood alcohol concentration. TAC levels were significantly higher in all patients with a blood alcohol concentration above 4‰ (*p* = 0.0178) compared with the control group. TOS levels were significantly lower in patients with a blood alcohol concentration above 4‰ compared with patients with a blood alcohol concentration approximating 2.1–4‰ (>4‰ vs. 2.1–3‰: *p* = 0.0083; >4‰ vs. 3.1–4‰: *p* = 0.009). The OSI was significantly lower in patients with a blood alcohol concentration above 4‰ compared with patients with a blood alcohol concentration below 4‰ (>4‰ vs. 0‰: *p* < 0.0001; >4‰ vs. 1.1–2‰: *p* = 0.0003; >4‰ vs. 2.1–3‰: *p* < 0.0001; >4‰ vs. 3.1–4‰: *p* = 0.0005) (Figure 4).

### 2.3. ROC Analysis

ROC analysis demonstrated that a urinary TOS concentration below 11.1 μmol/mg of protein (with the AUC for the TOS 0.74) allowed to distinguish between people with a blood alcohol concentration approximating 3.1–4‰ and above 4‰. Similarly, an OSI level below 4.75 μmol/mg of protein (AUC for OSI 0.8) allowed for the differentiation between people with a blood alcohol concentration approximating 3.1–4‰ and above 4‰ (Table 4).

### 2.4. Correlations

TOS blood concentration was positively correlated with blood TAC (r = 0.31, *p* < 0.0001) and blood OSI levels (r = 0.47, *p* < 0.0001). However, we recorded a negative correlation between the TAC and the OSI levels in the blood (r = −0.6, *p* < 0.0001).

The urine TAC was positively correlated with TOS (r = 0.15, *p* = 0.0293) and TAC (r = 0.2, *p* = 0.0032) levels in the blood. The TOS concentration in urine had a positive relationship with TOS (r = 0.17, *p* = 0.0091) and OSI (r = 0.15, *p* = 0.03156) levels in the blood. Moreover, TOS (r = 0.08, *p* = 0.2672) and OSI (r = 0.23, *p* = 0.001339) concentrations in the blood showed a positive relationship with the urine OSI.

Furthermore, the TAC concentration in the blood was negatively correlated with the OSI level (r = −0.24, *p* = 0.0005) in the urine, and the OSI concentration in the blood had a negative relationship with the TAC levels (r = −0.07, *p* = 0.3224) in the urine.

The blood TAC correlated positively with the blood alcohol (r = 0.48, *p* < 0.0001) and urine alcohol (r = 0.52, *p* < 0.0001) levels. TOS levels in the blood also correlated positively with the alcohol content in the blood (r = 0.35, *p* < 0.0001) and urine (r = 0.37, *p* < 0.0001). However, a negative relationship between the OSI concentration in the blood and blood alcohol (r = −0.29, *p* < 0.0001) and urine alcohol (r = −0.30, *p* < 0.0001) were shown.

**Figure 5:**
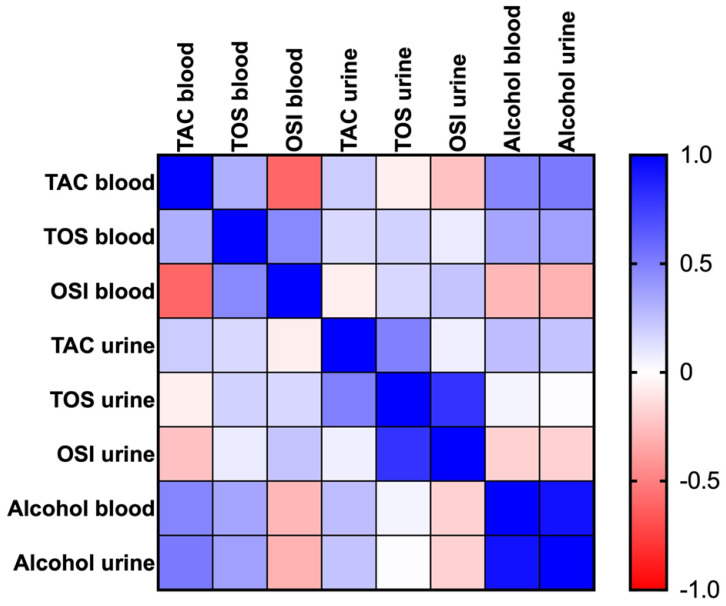
Heat map of correlations between blood and urine redox status biomarkers and alcohol concentrations. Abbreviations: TAC—total antioxidant capacity; TOS—total oxidative status; OSI—oxidative stress index.

Furthermore, the TAC concentration in the urine correlated positively with alcohol levels in the blood (r = 0.26, *p* < 0.0001) and urine (r = 0.23, *p* = 0.000433). We also recorded a negative relationship between the OSI concentration in the urine and blood alcohol (r = −0.18, *p* = 0.009) and urine alcohol (r = −0.18, *p* = 0.009668) (Figure 5).

## 3. Discussion

The results of our study offer significant insight into the utility of TAC, TOS, and the OSI as biomarkers of fatal outcomes associated with alcohol exposure. By examining those parameters in both blood and urine, we have identified critical differences in the circulating redox profile between individuals with alcohol dependency and alcohol poisoning.

Blood and urine TAC levels are significantly elevated in patients with both alcohol dependency and alcohol poisoning. Strengthening the antioxidant barrier might reflect an adaptive response to oxidative stress induced by alcohol metabolism [21,26,27,28]. Ethanol affects the liver’s Kupffer cells, which can eliminate alcohol metabolites. Ethanol biotransformation involves a higher activity of alcohol dehydrogenase (ADH), microsomal ethanol oxidizing system (MEOS), and CAT, which increases the production of acetaldehyde, reduced coenzymes (mainly NADH), and free radicals. Acetaldehyde and other alcohol metabolites form adducts with proteins, lipids, and DNA, stimulating the development of liver inflammation and matrix remodeling [21,23,29]. Studies in animal models have confirmed an increased oxidative stress in alcohol-exposed animals [21,30]. Elevated lipid, protein, and DNA oxidative damage has also been confirmed in alcohol-dependent human models [21,22]. Not surprisingly, strengthening the antioxidant barrier is one of the primary protective measures against ROS overproduction and oxidative stress [31]. The increase in the blood TAC level is more pronounced in the alcohol-poisoned group, suggesting that severe alcohol intoxication may trigger more robust compensatory mechanisms. Those findings align with the previous studies that report an altered antioxidant defense in people who use alcohol [21,22,32,33,34,35,36,37,38,39]. In individuals with alcohol dependency, SOD and GPx activity is reduced in erythrocytes but is also increased in serum/plasma, suggesting a complex and compartmentalized response to oxidative stress [20,22,33,35,40,41,42,43]. Additionally, higher ethanol consumption often increases CAT activity, reflecting an adaptive response to elevated hydrogen peroxide levels [22,35,40,44,45,46]. However, in the Erel method, the serum TAC of healthy subjects was composed of 52.9% from thiol groups of proteins, 33.1% from UA, 4.7% from vitamin C, 2.4% from bilirubin, 1.7% from vitamin E, and 5.2% from other antioxidants [14]. The urinary TAC is formed mainly by UA (75%) and proteins [13]. Although the contribution of antioxidants to TAC may vary in patients exposed to alcohol, the increase in the TAC levels may be due to the hyperuricemia that accompanies alcoholism [47]. Indeed, many studies have observed hyperuricemia in people who use alcohol [48,49,50,51,52]. UA is the end product of the purine metabolism, which is supplied to the body with food. UA plays a dual role as both an antioxidant and a pro-oxidant, significantly impacting redox homeostasis in blood and urine. As an antioxidant, UA is a potent scavenger of superoxide anions, hydroxyl radicals, and peroxynitrite, maintaining endothelial function and protecting against neurodegenerative diseases [53,54]. However, UA can also act as a pro-oxidant, forming aminocarbonyl radicals, urate free radicals, and urate hydroperoxide and exacerbating oxidative stress [53,55,56,57,58]. This pro-oxidant activity is linked to the pathogenesis of several diseases, including cardiovascular disorders and metabolic syndrome, which are prevalent in AUD individuals [53,59]. The balance between UA’s antioxidant and pro-oxidant roles is influenced by its concentration and the microenvironment, with higher levels leading to increased oxidative stress and associated AUD complications [60]. Alcohol consumption is the factor that significantly increases UA production [61,62]. Understanding and managing UA levels could be crucial in addressing oxidative stress-related conditions [53,63]. However, ethanol exposure also increases serum and biliary bilirubin levels [64,65,66,67,68], contributing to conditions like cholelithiasis due to increased unconjugated bilirubin secretion [69]. At the same time, a decrease in low-molecular-weight antioxidants like vitamins C [70,71,72] and E [73,74,75] is observed in patients with AUD. Not surprisingly, the assessment of total antioxidant capacity/activity is beneficial for assessing the antioxidant barrier. TAC is the resultant effect of various ROS scavengers reflecting the antioxidant protection against oxidative stress. Nevertheless, it is essential to remember that each available analytical technique is not free of limitations, such as evaluating only selected antioxidants [76,77,78,79,80].

TOS levels are elevated in both study groups relative to the controls. However, TOS levels are also significantly higher in patients with alcohol dependency compared with the alcohol-poisoned group, which may be explained by the weaker antioxidant defense (↑TAC) in the first group. TOS indicates the total content of oxidants in the biological sample; however, it mainly reflects the rate of lipid peroxidation [15,81]. Although lipids are easily oxidized, those reactions are very complex and occur in three steps: initiation, propagation, and termination. One of the main products of lipoperoxidation is malondialdehyde (MDA) [82]. The results of our study suggest that TOS may underscore the persistent oxidative burden caused by chronic alcohol consumption. Longer alcohol exposure may be associated with prolonged ROS overproduction, resulting in free radical-mediated damage to lipids. Indeed, in addition to the xenobiotic concentration, the time of exposure is a key determinant of its biological effects [83,84].

In our study, we observed elevated TAC and TOS levels in patients exposed to alcohol. The OSI integrates TOS with TAC and thus is used as a comprehensive assessment of redox status [11]. In our patients, the OSI was significantly lower in the alcohol-poisoned group compared with both the control and alcohol-dependent groups. That reduction may indicate an imbalance in redox homeostasis, in which the body’s antioxidant defense is intensively stimulated. Although further research is required, that hypothesis may be supported by a stronger correlation between blood alcohol content and TAC (r = 0.48, *p* < 0.0001) than TOS (r = 0.35, *p* < 0.0001). However, the dual nature of UA, which makes a significant contribution to TAC, should be kept in mind. The increase in TAC levels may be related to the pro-oxidant effect of UA [85].

According to the World Health Organisation (WHO), alcohol is associated with more than 200 diseases and injury conditions, and, unfortunately, the mortality rate related to alcohol consumption is higher than that caused by diabetes, tuberculosis, and HIV/AIDS [1]. As the number of people consuming alcohol continues to rise, it is not surprising that biomarkers of alcohol dependence are still being sought. Those biomarkers should provide objective information on alcohol consumption over time and thus apply to the diagnosis of alcohol-related diseases and assessment of treatment effectiveness. In our study, the analysis of diagnostic usefulness (ROC) has shown that the blood TAC discriminates between individuals with alcohol poisoning and alcohol dependency (AUC > 0.86) with the highest sensitivity (>82%) and specificity (>83%). However, when it comes to distinguishing people consuming alcohol from people not consuming alcohol, the blood TOS is a better indicator (AUC = 0.81, sensitivity = 75%, specificity = 83%). We also compared the diagnostic usefulness of redox biomarkers according to blood alcohol content at the time of death. The strongest associations with alcohol concentrations were recorded for the blood TAC (r = 0.48, *p* < 0.0001). In the case of ROC analysis, the blood TAC appeared to be the potentially best biomarker, distinguishing individuals with blood alcohol levels between 3.1 and 4‰ with a sensitivity and specificity of >85% (AUC = 0.89). Although we included a relatively large group of subjects carefully selected for concomitant diseases, further studies are needed to confirm the biomarker potential of circulating TAC, TOS, and OSI in AUD patients.

## 4. Materials and Methods

### 4.1. Patients

The Bioethics Committee of the Medical University of Bialystok, Poland has stated that, under current law, this study does not meet the definition of a medical experiment, as it will not be conducted on humans or involve biological material obtained from individuals for scientific purposes. Accordingly, their consent is not required (letter No. APK.002.2.2024). Due to the planned collection of material during medico-legal dissections conducted by forensic pathologists from Bialystok district units, permission was requested from the District Prosecutor’s Office in Bialystok, Poland, which issued its consent to conduct this study (letter No. 3001-4.070.1.2024).

This study examined three groups of deceased individuals. The first one (the alcohol-poisoned group) included 47 subjects (29 men and 18 women), ranging in age from 19 to 80 years, with a median age of 45, who died as a result of acute alcohol intoxication. All patients exhibited significant liver steatosis and brain atrophy. The second (alcohol-dependent) group, comprising 102 individuals with alcohol dependency (61 men and 41 women, aged 18 to 83, with a median age of 46.5 years), lost their lives due to suicide, traffic accidents, or other fatal events. In this group, both focal and diffuse liver steatosis were present, along with brain atrophy. The third (control) group consisted of 105 individuals who were not dependent on alcohol (65 men and 40 women) aged 19 to 88 years, with a median age of 52. Causes of death in this group were sudden, including brain injuries (72 cases, or 68.57%) and chest injuries (33 cases, or 31.43%). Death occurred instantly at the scene without any prolonged suffering. Individuals suffering from neurodegenerative diseases, autoimmune diseases (systemic scleroderma, rheumatoid arthritis), inflammatory diseases of the gastrointestinal tract, cardiovascular diseases, renal diseases, metabolic diseases (obesity, diabetes), and infectious diseases (HIV, HBV, HCV infection) were excluded from the control group and both study groups based on the interview collected from the family, medical documentation provided by the prosecutor, and autopsy findings. Table 5 presents the blood and urine alcohol concentrations of subjects from all groups.

### 4.2. Sample Collection

After being declared deceased, the bodies were immediately transported to the morgue. The bodies were kept refrigerated at 4 °C until the post-mortem examination. Family members confirmed there had been no drug abuse. In all patients, pathological changes in other organs, particularly in the heart and kidney, were ruled out. Conditions such as cancer and chronic inflammatory diseases and other health risk behaviors (e.g., smoking) were also excluded.

Biological samples were collected during a medico-legal autopsy 12 h post-mortem. Using a syringe, 5 mL of blood from the femoral vein and 5 mL of urine from the bladder were collected. The samples were immediately centrifuged at 3000× *g* for 20 min at 4 °C, and the resulting supernatant was preserved for further analysis.

Blood and urine ethanol concentrations were determined through the headspace gas chromatography method (HS-GC-FID). A Trace GC Ultra Chromatograph equipped with an FID detector and TriPlus automatic headspace injector (Thermo Electron Corporation, Waltham, MA, USA), was used. The ethanol standard curve ranged from 0.2 to 4.0‰ [18,86].

### 4.3. Redox Status

All reagents were obtained from Sigma-Aldrich (Burlington, MA, USA). Absorbance measurements were conducted utilizing the Tecan Infinite M200 PRO Multimode Microplate Reader (Tecan Group Ltd., Männedorf, Switzerland).

TAC was determined using a colorimetric method based on the procedure developed by Erel. This approach assesses the capacity of antioxidants in the sample to neutralize the 2,2-azino-bis-(3-ethylbenzothiazoline-6-sulfonate) (ABTS) cation radical (ABTS^•+^). TAC levels were calculated using a standard curve for Trolox (6-hydroxy-2,5,7,8-tetramethylchroman-2-carboxylic acid) and expressed as µm Trolox per mg of total protein. Each well received 200 µL of R1 solution (0.4 M acetate buffer, pH 5.8), 5 µL of the sample (blood, urine), and 20 µL of R2 solution (ABTS^•+^ in 30 mM acetate buffer, pH 3.6). Absorbance was initially measured (660 nm) before mixing R1 and R2, serving as the blank. Following mixing, absorbance readings were taken at 660 nm, both before and after a 5 min incubation period [14,87].

TOS was assessed using a colorimetric method following the procedure outlined by Erel. In this method, oxidants in the sample cause the oxidation of Fe^2+^ to Fe^3+^ ions, which are then detected by their reaction with xylenol orange. TOS levels were quantified using a calibration curve based on hydrogen peroxide. To each well, 225 µL of R1 solution (containing 150 μM xylenol orange, 140 mM NaCl, and 1.35 M glycerol in 25 mM H_2_SO_4_, pH 1.75), 35 µL of the sample (blood, urine), and 11 µL of R2 solution (comprising 5 mM ferrous ions and 10 mM o-dianisidine in 25 mM H_2_SO_4_) were added. The initial absorbance, serving as a blank, was recorded before mixing the reagents. Absorbance was measured at 560 nm and 800 nm (bichromatically), followed by immediate mixing of reagents and a second measurement after a 5 min incubation period [15,88].

The OSI was calculated as the ratio of TOS to TAC [87].

All measurements were carried out in duplicate and normalized to the total protein content. Protein concentration was determined using a bicinchoninic acid (BCA) assay, with a commercial kit from Thermo Scientific PIERCE BCA Protein Assay (Rockford, IL, USA).

### 4.4. Statistics

Statistical analysis was performed using GraphPad Prism 10 (GraphPad Software, La Jolla, CA, USA) and Past 4.13 (Øyvind Hammer). Due to the non-normal distribution of the data, the Kruskal–Wallis ANOVA test was applied. For more in-depth analysis, Dunn’s post hoc test and Spearman correlation were also employed. Results were expressed as medians (minimum/maximum) and percentiles. ROC analysis was used to evaluate the diagnostic effectiveness of TAC, TOS, and OSI, with the AUC and optimal cut-off points calculated for each parameter to ensure high sensitivity and specificity. A *p*-value of <0.05 was considered statistically significant in all tests.

## 5. Study Limitations

Assessing the redox homeostasis of the blood and urine is problematic and only partially reflects the phenomena occurring in the target organs (liver, kidneys, brain) [89]. TAC and TOS are comprehensive measures and do not provide information on specific antioxidants or ROS. The analyzed biomarkers may depend on many factors, such as diet, lifestyle, and health status, making interpretation of the data difficult. Temperature, pH, and sample storage conditions may also affect assay results. Although TAC, TOS, and OSI provide general information about the body’s redox status, their clinical interpretation may be difficult without additional testing [90,91,92,93,94,95]. Advanced techniques such as high-resolution mass spectrometry (HRMS) or metabolomic analyses would allow more detailed characterization of the oxidant and antioxidant molecules involved. The interpretation of blood and urinary redox status, heavily influenced by UA levels, requires caution, given UA’s dual role as an antioxidant and oxidant. Finally, the poor correlation between redox biomarkers in blood and urine may be due to changes in body organs or circulating fluids that occur post-mortem. Just a few minutes after death, autolysis and putrefaction occur under the influence of intracellular enzymes or bacteria [18,96]. Therefore, the impact of post-mortem processes on redox status cannot be excluded.

## 6. Future Perspectives

Future research should explore the specific ROS, antioxidants, and oxidative damage products contributing to the observed redox imbalance based on more sophisticated techniques (high-resolution mass spectrometry or metabolomics). They should also consider variables such as diet, smoking, lifestyle, health status, medication use, and pre-existing medical conditions. Finally, TAC, TOS, and OSI should be validated in more diverse populations to enhance clinical utility.

## 7. Conclusions

In conclusion, our study shows that blood TAC may be a potential biomarker of alcohol consumption. Blood demonstrates greater diagnostic utility than urine for assessing redox status in individuals exposed to alcohol. The increase in TAC over TOS may be an adaptive response to ROS overproduction related to alcohol exposure. However, further studies are needed, including a detailed assessment of redox homeostasis in circulating fluids and target organs (liver, kidney, and brain).

## Figures and Tables

**Table 1:** Receiver operating characteristic (ROC) analysis of redox biomarkers in blood differentiates between the control group (C), patients with alcohol dependency (AD), and patients with alcohol poisoning (AP). Abbreviations: AUC—area under the curve; CI—confidence interval; TAC—total antioxidant capacity; TOS—total oxidative status; OSI—oxidative stress index.

	TAC Blood			TOS Blood			OSI Blood		
	C vs. AD	C vs. AP	AD vs. AP	C vs. AD	C vs. AP	AD vs. AP	C vs. AD	C vs. AP	AD vs. AP
AUC	0.64	0.92	0.86	0.81	0.75	0.7	0.61	0.84	0.89
95% CI	0.57 to 0.72	0.87 to 0.97	0.79 to 0.92	0.75 to 0.88	0.65 to 0.84	0.62 to 0.79	0.52 to 0.69	0.77 to 0.91	0.83 to 0.95
Cut-off	>47.26	>86.2	>88.11	>5.34	>4.7	<6.18	>10.37	<6.32	<6.48
Sensitivity%	63.64	85.11	82.98	75.25	72.34	68.09	61.54	82.61	84.78
95% CI	53.82% to 72.44%	72.31% to 92.59%	69.86% to 91.11%	66.01% to 82.64%	58.24% to 83.06%	53.83% to 79.6%	51.27% to 70.87%	69.28% to 90.91%	71.78% to 92.43%
Specificity%	61.9	91.43	83.84	83.33	70.59	66.34	60.22	80.22	86.02
95% CI	52.35% to 70.62%	84.51% to 95.43%	75.35% to 89.8%	74.92% to 89.33%	61.13% to 78.55%	56.67% to 74.8%	50.05% to 69.57%	70.89% to 87.11%	77.54% to 91.65%

**Table 2:** Receiver operating characteristic (ROC) analysis of redox biomarkers in blood differentiates between subjects with varied blood alcohol concentrations: 0‰; 1.1–2‰; 2.1–3‰; 3.1–4‰; above 4‰. Abbreviations: AUC—area under the curve; CI—confidence interval; TAC—total antioxidant capacity; TOS—total oxidative status; OSI—oxidative stress index.

	TAC Blood				TOS Blood				OSI Blood			
	0‰ vs. 1.1–2‰	1.1–2‰ vs. 2.1–3‰	2.1–3‰ vs. 3.1–4‰	3.1–4‰ vs. >4‰	0‰ vs. 1.1–2‰	1.1–2‰ vs. 2.1–3‰	2.1–3‰ vs. 3.1–4‰	3.1–4‰ vs. >4‰	0‰ vs. 1.1–2‰	1.1–2‰ vs. 2.1–3‰	2.1–3‰ vs. 3.1–4‰	3.1–4‰ vs. >4‰
AUC	0.69	0.58	0.52	0.89	0.83	0.54	0.59	0.62	0.62	0.56	0.57	0.85
95% CI	0.59 to 0.81	0.44 to 0.72	0.38 to 0.67	0.8 to 0.97	0.72 to 0.94	0.4 to 0.67	0.46 to 0.72	0.48 to 0.77	0.49 to 0.74	0.42 to 0.7	0.42 to 0.71	0.73 to 0.96
Cut-off	>50.14	<58.68	<50.44	>85.16	>5.1	>8.14	<7.21	<6.01	>10.76	>11.94	<11.93	<6.31
Sensitivity %	67.74	60.98	53.57	86.96	80	50	60	63.04	62.07	54.05	53.85	82.22
95% CI	50.14% to 81.43%	45.73% to 74.34%	35.81% to 70.47%	74.33% to 93.88%	62.69% to 90.49%	35.53% to 64.47%	42.32% to 75.41%	48.6% to 75.48%	44% to 77.31%	38.38% to 68.96%	35.46% to 71.24%	68.67% to 90.71%
Specificity %	68.57	58.06	53.66	85.71	80.39	53.33	57.14	63.33	62.37	55.17	54.05	80.77
95% CI	59.17% to 76.66%	40.77% to 73.58%	38.75% to 67.94%	68.51% to 94.3%	71.65% to 86.93%	36.14% to 69.77%	42.21% to 70.88%	45.51% to 78.13%	52.21% to 71.54%	37.55% to 71.59%	38.38% to 68.96%	62.12% to 91.49%

**Table 3:** Receiver operating characteristic (ROC) of redox biomarkers in urine differentiates between the control group (C), patients with alcohol dependency (AD), and patients with alcohol poisoning (AP). Abbreviations: AUC—area under the curve; CI—confidence interval; TAC—total antioxidant capacity; TOS—total oxidative status; OSI—oxidative stress index.

	TAC Urine			TOS Urine			OSI Urine		
	C vs. AD	C vs. AP	AD vs. AP	C vs. AD	C vs. AP	AD vs. AP	C vs. AD	C vs. AP	AD vs. AP
AUC	0.6	0.68	0.55	0.64	0.59	0.72	0.58	0.76	0.84
95% CI	0.52 to 0.69	0.56 to 0.79	0.44 to 0.66	0.56 to 0.72	0.48 to 0.7	0.62 to 0.81	0.49 to 0.66	0.67 to 0.84	0.77 to 0.91
Cut-off	>1.4	>1.49	>1.56	>11.09	<8.02	<10.26	>7.14	<4.76	<5.22
Sensitivity %	56.84	62.16	59.46	58.06	56.41	64.1	54.12	72.22	77.78
95% CI	46.81% to 66.34%	46.1% to 75.94%	43.49% to 73.65%	47.91% to 67.58%	40.98% to 70.7%	48.42% to 77.26%	43.58% to 64.3%	56.01% to 84.15%	61.92% to 88.28%
Specificity %	56.38	63.83	52.63	61.05	57.89	61.29	53.85	69.23	76.47
95% CI	46.3% to 65.96%	53.75% to 72.82%	42.69% to 62.37%	51% to 70.25%	47.85% to 67.33%	51.13% to 70.55%	43.66% to 63.72%	59.13% to 77.77%	66.43% to 84.22%

**Table 4:** Receiver operating characteristic (ROC) of redox biomarkers in urine differentiates between subjects with varied blood alcohol concentrations: 0‰; 1.1–2‰; 2.1–3‰; 3.1–4‰; above 4‰. Abbreviations: AUC—area under the curve; CI—confidence interval; TAC—total antioxidant capacity; TOS—total oxidative status; OSI—oxidative stress index.

	TAC Urine				TOS Urine				OSI Urine			
	0‰ vs. 1.1–2‰	1.1–2‰ vs. 2.1–3‰	2.1–3‰ vs. 3.1–4‰	3.1–4‰ vs. >4‰	0‰ vs. 1.1–2‰	1.1–2‰ vs. 2.1–3‰	2.1–3‰ vs. 3.1–4‰	3.1–4‰ vs. >4‰	0‰ vs. 1.1–2‰	1.1–2‰ vs. 2.1–3‰	2.1–3‰ vs. 3.1–4‰	3.1–4‰ vs. >4‰
AUC	0.58	0.51	0.58	0.5	0.58	0.56	0.5	0.74	0.55	0.56	0.56	0.8
95% CI	0.45 to 0.72	0.37 to 0.65	0.44 to 0.72	0.36 to 0.65	0.46 to 0.7	0.42 to 0.7	0.36 to 0.64	0.62 to 0.86	0.44 to 0.67	0.41 to 0.71	0.42 to 0.71	0.69 to 0.92
Cut-off	>1.41	<1.51	>1.6	>1.67	>9.8	>11.85	<13.91	<11.1	>7.14	>7.22	<7.21	<4.75
Sensitivity %	57.14	53.85	55.17	50	55.56	57.89	44.83	65.79	53.85	54.55	51.85	71.43
95% CI	39.07% to 73.49%	38.57% to 68.43%	37.55% to 71.59%	34.47% to 65.53%	37.31% to 72.41%	42.19% to 72.15%	28.41% to 62.45%	49.89% to 78.79%	35.46% to 71.24%	37.99% to 70.16%	33.99% to 69.26%	54.95% to 83.67%
Specificity %	56.38	50	56.41	48.28	51.58	59.26	42.11	62.07	53.85	53.85	54.55	74.07
95% CI	46.3% to 65.96%	32.63% to 67.37%	40.98% to 70.7%	31.39% to 65.57%	41.67% to 61.37%	40.73% to 75.49%	27.85% to 57.81%	44% to 77.31%	43.66% to 63.72%	35.46% to 71.24%	37.99% to 70.16%	55.32% to 86.83%

**Table 5:** Blood and urine alcohol concentration in individuals from the control group, patients with alcohol dependency, and patients with alcohol poisoning.

	Control		Alcohol Dependence		Alcohol Poisoning	
	Blood	Urine	Blood	Urine	Blood	Urine
Median	0‰	0‰	2.6‰	1.7‰	4.4‰	4.1‰
Minimum	0‰	0‰	1‰	0.5‰	4‰	4‰
Maximum	0‰	0‰	3.8‰	3.8‰	6.2‰	5.5‰

## Data Availability

The data presented in this study are available on request from the corresponding author due to reasonable request.
